# Clinicians’ Experiences of Implementing a Telerehabilitation Toolkit During the COVID-19 Pandemic: Qualitative Descriptive Study

**DOI:** 10.2196/44591

**Published:** 2023-03-10

**Authors:** Sarah Munce, Angie Andreoli, Mark Bayley, Meiqi Guo, Elizabeth L Inness, Ailene Kua, McKyla McIntyre

**Affiliations:** 1 KITE Research Institute Toronto Rehabilitation Institute University Health Network Toronto, ON Canada; 2 Department of Occupational Science and Occupational Therapy University of Toronto Toronto, ON Canada; 3 Rehabilitation Sciences Institute University of Toronto Toronto, ON Canada; 4 Institute of Health Policy, Management and Evaluation Toronto, ON Canada; 5 Department of Physical Therapy University of Toronto Toronto, ON Canada; 6 Toronto Rehabilitation Institute University Health Network Toronto, ON Canada; 7 Division of Physical Medicine and Rehabilitation Department of Medicine University of Toronto Toronto, ON Canada

**Keywords:** telerehabilitation, implementation, toolkit, COVID-19, qualitative, clinician

## Abstract

**Background:**

Although the COVID-19 pandemic resulted in a rapid implementation and scale-up of telehealth for patients in need of rehabilitation, an overall slower scaling up to telerehabilitation has been documented.

**Objective:**

The purpose of this study was to understand experiences of implementing telerehabilitation during the COVID-19 pandemic as well as using the Toronto Rehab Telerehab Toolkit from the perspective of rehabilitation professionals across Canada and internationally.

**Methods:**

The study adopted a qualitative descriptive approach that consisted of telephone- or videoconference-supported interviews and focus groups. Participants included rehabilitation providers as well as health care leaders who had used the Toronto Rehab Telerehab Toolkit. Each participant took part in a semi-structured interview or focus group, lasting approximately 30-40 minutes. Thematic analysis was used to understand the barriers and enablers of providing telerehabilitation and implementing the Toronto Rehab Telerehab Toolkit. Three members of the research team independently analyzed a set of the same transcripts and met after each set to discuss their analysis.

**Results:**

A total of 22 participants participated, and 7 interviews and 4 focus groups were included. The data of participants were collected from both Canadian (Alberta, New Brunswick, and Ontario) and international sites (Australia, Greece, and South Korea). A total of 11 sites were represented, 5 of which focused on neurological rehabilitation. Participants included health care providers (ie, physicians, occupational therapists, physical therapists, speech language pathologists, and social workers), managers and system leaders, as well as research and education professionals. Overall, 4 themes were identified including (1) implementation considerations for telerehabilitation, encompassing 2 subthemes of “infrastructure, equipment, and space” and “leadership and organizational support”; (2) innovations developed as a result of telerehabilitation; (3) the toolkit as a catalyst for implementing telerehabilitation; and (4) recommendations for improving the toolkit.

**Conclusions:**

Findings from this qualitative study confirm some of the previously identified experiences with implementing telerehabilitation, but from the perspective of Canadian and international rehabilitation providers and leaders. These findings include the importance of adequate infrastructure, equipment, and space; the key role of organizational or leadership support in adopting telerehabilitation; and availing resources to implement it. Importantly, participants in our study described the toolkit as an important resource to broker networking opportunities and highlighted the need to pivot to telerehabilitation, especially early in the pandemic. Findings from this study will be used to improve the next iteration of the toolkit (Toolkit 2.0) to promote safe, accessible, and effective telerehabilitation to those patients in need in the future.

## Introduction

Rehabilitation aims to enhance and restore functional ability, independence, and quality of life for those with physical, cognitive, and communication impairments or disabilities. Access to rehabilitation can be especially challenging for individuals with disabilities in rural communities as well as those who are less able to attend in-person therapy due to distance, transportation, financial resources, and mobility challenges [1-3]. Ongoing rehabilitation often requires therapy over many sessions, which can be challenging for maintaining continuity of care when travel to appointments is required [4]. Ensuring equitable access to rehabilitation services by identifying, targeting, and removing barriers faced by underserved and vulnerable populations has been recognized as a key component of a comprehensive rehabilitation system [5].

Telerehabilitation has been increasingly used as a means to address these challenges (ie, reducing the burden of travel time and related fatigue, improving access to care, and continuity of care) [6]. During the COVID-19 pandemic, telerehabilitation has been critical to providing ongoing care for those people living with impairments or disabilities [4]. Telerehabilitation is a branch of telemedicine that uses telecommunication technologies to deliver rehabilitation services synchronously or asynchronously to patients at a distance [7]. Specifically, telerehabilitation encompasses diagnosing, evaluating, and managing health care for persons with physical, cognitive, or social impairment and disability [7]. Telerehabilitation has been shown to be both feasible and effective in chronic heart failure and coronary artery disease [8], stroke [9], multiple sclerosis [10], and spinal cord injuries [11].

Although the COVID-19 pandemic resulted in a rapid implementation and scale-up of telehealth [12,13], for patients in need of rehabilitation, an overall slower scaling up to telerehabilitation has been documented [9]. This has brought to the forefront a need for rehabilitation researchers and clinicians to better understand how to deliver effective telerehabilitation services in ways that are safe to patients.

To address these challenges, our team developed the Toronto Rehab Telerehab Toolkit. The telerehabilitation implementation team at Toronto Rehab included practice leaders, program service managers, a researcher, and a physician, who were engaged throughout all phases of program development, implementation, and evaluation. The toolkit was then developed through consultation and co-development with health care providers, leaders, patients, and caregivers, with the aim of continuously evolving through user feedback and experience as a telerehabilitation community. The goal of this toolkit was to provide a guiding framework to improve access to rehabilitation through telerehabilitation and to share our knowledge, insights, and lessons learned from the early phases of the pandemic. The toolkit contains resources and processes around 4 implementation domains: getting started, preparing patients and carers, implementing virtual rehab, and evaluation and monitoring [14]. Thus, the purpose of this study was to understand experiences of implementing telerehabilitation during the COVID-19 pandemic as well as using the Toronto Rehab Telerehab Toolkit from the perspective of rehabilitation professionals across Canada and internationally.

## Methods

### Study Design

This study adopted a qualitative descriptive approach that consisted of telephone- or web-based (ie, Microsoft Teams) interviews and focus groups. Previous research has demonstrated the viability of other videoconferencing platforms (ie, Zoom) for qualitative data collection because of its ease of use, cost-effectiveness, data management options, and security features [15]. A qualitative descriptive design is a well-accepted approach for studying topics about which little is known and providing practical solutions that are relevant to policy makers and health care practitioners [16,17]. Telephone- or web-based interviews and focus groups were selected because of the geographic dispersion of the study participants. We followed the Consolidated Criteria for Reporting Qualitative Research checklist [18] for the reporting of the study. This checklist promotes the reporting of the important components of a qualitative study, including the research team, methods, context, results, and interpretations.

### Recruitment

Participants included rehabilitation providers (eg, physicians, occupational therapists, and physical therapists) as well as health care leaders who had provided telerehabilitation and implemented the Toronto Rehab Telerehab Toolkit. Participants were contacted by email about their willingness to participate in the interview and focus group if they first consented to being contacted for this purpose when they requested a copy of the toolkit. Purposive sampling (ie, maximum variation) [19] was used to ensure diversity in geography, type of rehabilitation center, and rehabilitation population. Participants were recruited between January and August 2021. Recruitment ceased when a discussion and review of the responses revealed that saturation had been achieved [20].

### Data Collection

Each participant took part in a semistructured telephone- or web-based interview or focus group, lasting approximately 30-40 minutes. Members of the research team (SM and AA) conducted the interviews and focus groups. The interview and focus group guide consisted of semistructured, open-ended questions and was pilot-tested with 1 leader and 1 provider, and it was refined in response to feedback. Probes or recursive questioning were used to explore issues in greater depth and to verify understanding of the information being collected [19]. The probes were revised and refined as data collection progressed to establish saturation [19,21]. The complete list of questions is included in Multimedia Appendix 1. No repeat focus groups or interviews were conducted; all were digitally recorded. The recordings were transcribed verbatim for data analysis by a professional transcriptionist. These transcripts were not returned to participants for comments or corrections. Field notes were made during or after the interviews and focus groups.

### Data Analysis

Thematic analysis as described by Braun and Clark [22] was used to understand the barriers and enablers of providing telerehabilitation and implementing the Toronto Rehab Telerehab Toolkit. Three members of the research team (SM, AA, and MM) independently coded a set of the same transcripts and met after each set to discuss their codes. During these meetings, codes were discussed, and discrepancies were resolved until agreement of the coded transcripts was reached. After the first meeting, an initial codebook was established and applied to the new set of transcripts. The codebook was revised as themes were identified. SM is a scientist and has a PhD in Health Services Research as well as expertise in knowledge translation. She has approximately 14 years of experience conducting qualitative research. AA is a physiotherapist with expertise in implementation science, patient experience, and neurological rehabilitation. She has 16 years of experience with qualitative methods and methodologies, including conducting interviews and focus groups. MM is a physiatrist (ie, MD) with expertise in stroke, brain injury, and rehabilitation research. Disagreements or discrepancies around codes, themes, and subthemes were resolved by a group discussion and reference to the original transcripts. The themes were not shared with participants due to feasibility considerations.

### Ethical Considerations

This project was reviewed by the Quality Improvement Review Committee of University Health Network. The nature of the project was deemed as quality assurance or quality improvement, as defined in Tri-Council Policy Statement V.2, and the project was provided with a Research Ethics Board exemption.

## Results

### Description of Participants

A total of 22 participants participated, and 7 interviews and 4 focus groups were included. The data of participants from both Canadian (Alberta, New Brunswick, and Ontario) and international sites (Australia, Greece, and South Korea) were collected (Table 1). A total of 11 sites were represented (Table 2), 5 of which focused on neurological rehabilitation. Participants included health care providers (ie, physicians, occupational therapists, physical therapists, speech language pathologists, and social workers), managers and system leaders, as well as research and education professionals. There were no refusals to participate or dropouts.

**Table 1:** Description of participants by profession.

Participants by profession	Values, n (%)
Providers (occupational therapists, physical therapists, speech language pathologists, and social workers)	7 (31.8)
Physicians	3 (13.6)
Managers and leaders	6 (27.3)
System leaders	2 (9.1)
People with lived experiences	1 (4.5)
Research and education	3 (13.6)

**Table 2:** Description of type of rehab.

Type of rehab	Values, n (%)
Neurological	5 (45.5)
Cardiac	2 (18.2)
General	2 (18.2)
Private practice (neurological focus)	1 (9.1)
Long-term care	1 (9.1)

### Overview of Themes

Overall, 4 themes were identified including (1) implementation considerations for telerehabilitation, encompassing 2 subthemes of “infrastructure, equipment, and space” and “leadership and organizational support”; (2) innovations developed as a result of telerehabilitation; (3) the toolkit as a catalyst for implementing telerehabilitation; and (4) recommendations for improving the toolkit. The implementation considerations subthemes could be considered barriers or facilitators to implementing telerehabilitation depending on their presence or absence. Some representative quotations were identified and selected from the transcripts to illustrate the themes.

### Implementation Considerations for Telerehabilitation

Within the theme of implementation considerations, presence of adequate infrastructure, equipment, and space was described as a facilitator to implementing telerehabilitation. Participants emphasized the importance of extra computers; training for platforms, such as Zoom and Skype, to conduct virtual care; and technological support at their organizations. Some participant perspectives are as follows:

And it took them [providers] a while to figure that out. And I have to say that our teams were amazingly innovative and really reached out with as many people as possible to try and flex these programs like Zoom and Skype to the max. When they were using [virtual] breakout rooms, I have to say they are pretty resilient in trying to figure that out. So, it worked for some teams.

Site 1

Well, we actually now are spending probably our first, like, our first class session is just basically, an introduction to Zoom. Some patients don’t even have an email, so if they have an email, we can get them set up with an email, we tell them that’s all you need,…an email link. But that first session is usually a challenge.

Site 2

The prioritization of space for telerehabilitation was also seen as a facilitator to care, as the following quote represents:

They [providers] feel that the clinic room has enough space to be able to do that, but they need the equipment to be able to outfit it. So, we’ve put forward that we would like five multipurpose clinic rooms that will allow either in-person or virtual.

Site 1

Conversely, participants also described a lack of infrastructure, equipment, and space as barriers to implementing telerehabilitation. Specifically, participants described patients’ own lack of equipment or internet access as barriers to telerehabilitation and the difficulties of finding dedicated and appropriate space for virtual care. Below are some quotes illustrating this theme:

Some patients don’t have a blood pressure machine, some patients just can’t do it, some patients don’t have the ability to figure out the Six-Minute Walk [Test]. There’s a really nice…app, but if you don’t have a cell phone,…It’s hard for patients.

Site 2

Not all of our patients have internet access, not all of our patients have devices, they don’t have computer access, they just don’t, and some of our patients aren’t in the city setting, it’s remote.

Site 2

Leadership and organizational support was another subtheme of implementation considerations for telerehabilitation. Participants described its presence and absence as both a facilitator and barrier to implementing telerehabilitation, such as the following quotes:

So, our facility ramped up the access to equipment, expanded the use of our personal devices to be able to support virtual.

Site1

Imagine that we have to find ourselves the personal computer or the camera to do these things. Sometimes, [clinician name] and I, we brought, ourselves, our own personal laptops to do this. We even had to persuade the [names a leadership role] of the hospital to allow us to do that.

Site 3

### Innovations Developed as a Result of Telerehabilitation

Participants also described innovations that resulted from implementing telerehabilitation during the pandemic. Some of these included interprofessional assessments (eg, performed by both an occupational therapist and a physical therapist), which were described as especially helpful for complex patients. Another site described the development of a virtual hospital. Finally, another participant described their site’s heightened use of home pulse oximetry as a result of implementing telerehabilitation as one way for patients to track their outcomes at home.

### Toolkit as a Catalyst for Implementing Telerehabilitation

Participants often described the toolkit as a device in and of itself to reach out to other clinicians about telerehabilitation (ie, establishing a community of practice), such as the following perspective: “I think it’s a great engagement tool for planning when talking with clinicians” (Site 5). Participants also indicated that the toolkit was helpful to demonstrate the importance of telerehabilitation to their organizations, especially during the early stages of the pandemic. For example, at the onset of the pandemic, one site showed the toolkit to their leadership team and indicated “…look at what they are doing at Toronto Rehab. They are innovating next door. We need to do this” (Site 6). Participants at this same site indicated that the toolkit also provided them with credibility to continue rehabilitation during this early stage and view rehabilitation as an essential service.

### Recommendations for the Toolkit

Lastly, participants also offered specific recommendations for improving the toolkit. These included adding practical content, such as diagrams, videos, tips of the week, and patient stories. Participants also suggested including specific information on how to conduct virtual assessments, how to address liability, and prompting sites to tailor the content of the toolkit to their own contextual needs.

## Discussion

The purpose of this qualitative descriptive study was to understand experiences of implementing telerehabilitation during the COVID-19 pandemic as well as using the Toronto Rehab Telerehab Toolkit from the perspective of rehabilitation professionals across Canada and internationally.

Overall, 4 themes were identified including implementation considerations for telerehabilitation; innovations developed as a result of telerehabilitation; the toolkit as a catalyst for implementing telerehabilitation; and recommendations for improving the toolkit.

We identified 2 key implementation subthemes [23] for telerehabilitation, which were described as both barriers and facilitators. One subtheme was infrastructure, equipment, and space. This barrier has been previously reported on by both Negrini and colleagues [24] and Jafni [23], whereby limited technical resources, a dearth of devices, and slow internet bandwidth on the part of patients were identified as key barriers to telerehabilitation. Barriers with respect to infrastructure and equipment can be exacerbated by the potentially high levels of physical, emotional, and cognitive efforts needed to be engaged in telerehabilitation [22]. Indeed, participants in our study described some of the difficulties that older or more complex patients experience while participating in telerehabilitation and the critical and multidimensional roles that caregivers play in assisting in meaningful participation. Similarly, low expertise in using specific hardware or software on the part of health care providers has been previously identified as a barrier to telerehabilitation implementation [21]. In our study, participants indicated that a lack of technical expertise could be mitigated by dedicated IT support for the specific purpose of telerehabilitation and the necessary organizational leadership. Kreider and colleagues [4] also noted the crucial role of administrative support from rehabilitation management in terms extra and quiet rooms as well as computers and accessories needed to ensure patients’ privacy during telerehabilitation sessions.

The critical role of organizational and leadership support was also reported in the study by Kreider and colleagues [4]. For example, in studying providers’ shift to telerehabilitation at the US Veterans Health Administration during COVID-19, Kreider and colleagues [4] identified a “willingness to give telerehabilitation a chance” as a “key ingredient” to implementing telerehabilitation. The authors noted that across a variety of levels (ie, patient, provider, or leadership), this willingness, in addition to making adjustments and persisting with the use of available technologies, was essential to successfully transitioning to telerehabilitation services during the pandemic. Specifically, the authors described the importance of administrative support by medical leadership and rehabilitation managers to lead these efforts. In our study, some participants noted that the existence of the toolkit acted as a catalyst for implementing telerehabilitation in that it provided credence to characterizing rehabilitation as an essential service and implementing telerehabilitation, particularly early in the pandemic.

Participants in our study also described how innovations have been accelerated because of the use of telerehabilitation. The COVID-19 pandemic heightened the imperative for clinicians and researchers to better understand the practicalities of delivering telerehabilitation services in ways that are both safe and effective. The need for practical guidance in implementing telerehabilitation is indeed exemplified by the breadth of this practical guidance available through web-based sources [25-29]. As a result, we developed the Toronto Rehab Telerehab Toolkit, which provided this consolidated practical guidance, and as identified by our participants, brokered networking opportunities with other clinicians (locally, provincially, and nationally), leading to the establishment of a community of practice in some cases. Some study participants also described the increased use of remote technologies that were available because of telerehabilitation, including pulse oximetry. A systematic review of the effectiveness and safety of pulse oximetry in remote monitoring of patients with COVID-19 has supported its safety and usefulness for identifying the risk of deterioration and the need for advanced care [30].

Finally, participants provided a number of recommendations to improve the next version of the toolkit. These included very practical additions such as “tips of the week” for providers and using patient stories to share learnings and accelerate change. Participants also suggested including specific information on how to conduct virtual assessments and how to address concerns about potential liability. Similarly, Kreider and colleagues [4] described that providers had to change their approaches when conducting clinical assessments via telerehabilitation, including initially being challenged to find creative and innovative solutions to address the move from in-person, hands-on clinical assessment methods and measurement tools. Some participants detailed how they mitigated these challenges by shifting the assessment to a more functional focus with diligent clinical observation. Kreider and colleagues [4] also highlighted issues of patient safety with telerehabilitation and the critical role that family members played in ensuring safety during these remote sessions. The authors have used their own findings to develop a list of strategies and supports for telerehabilitation sessions during the chart review and scheduling, setting up or preparation, assessment and intervention planning, and during the session, as well as administrative supports. The specific, identified recommendations in this study will be incorporated into the next version of our toolkit.

We acknowledge some limitations in our study. We likely had a selection bias in terms of the participants who were interviewed in our study. Participants who had more positive experiences with telerehabilitation and the toolkit were more likely to participate. Similarly, only providers and health care leaders who had implemented the Toronto Rehab Telerehab Toolkit participated. Furthermore, none of the study participants were from rural rehabilitation sites. It is likely that providers at these sites would have had different experiences with telerehabilitation and the toolkit compared to clinicians from urban centers. At the same time, our study had a number of strengths in terms of demonstrating multiple aspects of trustworthiness including peer debriefing (credibility); a description of the study sample, although more detailed information about our participants could have been obtained (transferability); independent review of the data to arrive at codes and themes (dependability); and decision trails between data and interpretation (confirmability) [31].

Findings from this qualitative study confirm some of the previously identified experiences with implementing telerehabilitation but from the perspective of Canadian and international rehabilitation providers and managers. These findings include the importance of adequate infrastructure, equipment, and space as well as the key role of organizational and leadership support in adopting telerehabilitation and availing resources to implement it. Importantly, participants in our study described the toolkit as an important resource to broker networking opportunities and highlight the need to pivot to telerehabilitation, especially early in the pandemic. Specific recommendations gleaned from this study will be used to improve the next iteration of the toolkit (Toolkit 2.0) to promote safe, accessible, and effective telerehabilitation to those patients in need into the future.

## Appendix

Multimedia Appendix 1 List of questions.

## Data Availability

The data sets generated or analyzed during this study are not publicly available, as this was not outlined in the quality improvement application, but they are available from the corresponding author on reasonable request.
