# Supplementary material for: Clinicians’ Experiences of Implementing a Telerehabilitation Toolkit During the COVID-19 Pandemic: Qualitative Descriptive Study
Source: JMIR Rehabil Assist Technol. 2023 Mar 10;10:e44591. doi: 10.2196/44591 (PMC10039414; doi:10.2196/44591)
Supplement: Multimedia Appendix 1 [file rehab_v10i1e44591_app1.pdf]

**Interviewers:** **Date of interview:**

**Facilitator's guide for post-implementation interview**
**The TR- Telerehab Toolkit**

| |
|---|
| **Name:**<br>**Organization:**<br>**Area of practice:**<br>**Role:**<br>**What best describes your use of the toolkit:** |

**Introduction (2 min)**
Thanks for agreeing to participate in today's discussion. We are interested in understanding more about your implementation of virtual care over the past year or so with the goal to improve our Telerehab Implementation Toolkit here at Toronto Rehab.

Our conversation today will take about 30 minutes and your responses will be themed with other partner organizations who are also implementing virtual care.

By understanding your virtual care journey, as well as how the Toolkit may or may not have played a role, or goal is to enhance future iterations of the toolkit. With your ok, we will audio record this session, so we don't miss anything. I am turning on the audio recorder now.

**Section I: Experiences implementing virtual therapy within your setting (8 min)**

1. Could you share with us some of your experiences in implementing virtual care within your setting? We'd love to hear some of the highs and the lows in the evolution of your program.

**Probe**

- What were some of the factors that have enabled or facilitated implementation of virtual care in your setting?
- What were some of the primary challenges that you encountered when implementing virtual care? What *still* needs to happen for virtual care implementation to be successful?

**Section II: Role of the TR Toolkit in supporting virtual care (8 min)**

2. Building on these high and lows, can you share with us how the toolkit may have helped to address some of these issues?

**Probe**

- Given some of the enablers you shared, how did the toolkit support or leverage implementation of virtual care, if at all?
- How did the toolkit help to overcome or mitigate any of the challenges?

**Additional probes for Section II**
**Clinical or individual level factors**

- Staff knowledge, skills or self-efficacy in implementing virtual care (e.g., preparation of patients and caregivers to participate in virtual care, becoming familiar with technology)

**Patient and carer-specific factors**

- Patient-specific - Patient complexity and level of impairment, language barriers, technology barriers (equipment, internet speed)
- Caregiver-specific - Support to safely and meaningfully participate in care

**Workflows and processes** - Who needed to do what differently?

**Environment factors**

- Equipment and technology
- Organizational culture - Leadership support and expectations? Engagement?

**Section III: Improving the TR Telerehab Toolkit to support future implementation (7 min)**

This final section seeks your ideas to help inform the next iteration of the toolkit.

**3. a)** Was there any content missing from the toolkit? What else would have been helpful to better support your implementation?

**b)** We would like to devote a section in our next iteration to telerehab delivery, with practical tips, tools and ideas for providers. Does that resonate? What content would be most useful to you?

**c)** Is there anything else you would like to share with us? Is there anyone else you feel we should talk to?

Thank you for your time and insights!

**Field notes:**
